# Cryopreservation of Sperm: A Review

**DOI:** 10.7759/cureus.31402

**Published:** 2022-11-12

**Authors:** Gururaj M Borate, Ajay Meshram

**Affiliations:** 1 Department of Anatomy, Jawaharlal Nehru Medical College, Datta Meghe Institute of Medical Sciences, Wardha, IND; 2 Department of Biochemistry, Jawaharlal Nehru Medical College, Datta Meghe Institute of Medical Sciences, Wardha, IND

**Keywords:** cryopreservation, sperm, fertility, cancer, sperm bank

## Abstract

Sperm cryopreservation has been used as a sperm preservation solution for infertility issues faced by men undergoing cancer treatment for over 40 years. Recent developments in sperm cryopreservation and its wide variety of therapeutic uses are discussed in this article, which offers a succinct and up-to-date overview of the relevant literature. Recently, sperm cryopreservation has been employed for a wider variety of therapeutic purposes. As a result, sperm freezing is becoming available to a wider variety of patients, which requires more specialized personnel and increases overhead expenses. While sperm cryopreservation before cancer treatment is accessible in many countries, oncology doctors' referral rates and patient participation with cryopreservation services have been observed to be poor. In addition, there are still moral concerns with sperm banking, including whether or not donors' identities should be protected and whether or not a deceased person's sperm should be used after his or her death. This article discusses the recent developments in sperm cryopreservation technology and the moral questions that have arisen around this practice, with an eye toward how a deeper knowledge of these concerns can help more people get access to treatments that might help preserve their fertility. A sperm bank will notify clients about the screenings it does and the background information it gathers on individual donors to guarantee the safety and quality of the sperm they get. The viability and quantity of viable sperm in a thawed sample are often guaranteed by a sperm bank. They will look for very fertile males who can donate sperm that can withstand the freezing and thawing procedure. In many cases, sperm banks advertise their samples as having a certain number of viable sperm per milliliter, and they may provide many sample types for various applications (intracytoplasmic sperm injection and intrauterine insemination).

## Introduction and background

A sperm bank, also known as a semen bank or cryobank, is a business that acquires, banks, and distributes human sperm. Donor sperm is collected and sold by males in the fertility industry. A pregnancy or pregnancies can be sought by means other than a sexual relationship, and sperm is acquired for or on behalf of such purposes [1]. Donor sperm refers to sperm that has been sold by a sperm donor. The term "sperm bank" may refer to either an independent business that provides donor sperm to people or fertility clinics, or it can refer to a facility that is operated by a doctor-operated clinic or other medical organizations primarily or entirely for the benefit of their patients or clients. It is possible to become pregnant with artificial insemination using donor sperm, which has the same success rate as having sex naturally. It is a kind of third-party reproduction since donor sperm is used instead of that of the sperm recipient's partner. In the 21st century, artificial insemination using a donated sperm sample from a place used to store sperm or the sperm bank has been most widely utilized by persons without a male partner, i.e., single women and women in same-sex partnerships [2]. Generally speaking, there are age and health background checks that a sperm donor must pass [3]. In the United States, sperm banks are under the observation of the Food and Drug Administration's Human Cell and Tissue or Cell and Tissue Bank Product regulations. In addition to federal laws, several states have their own regulations [4]. According to the European Union (EU) Tissue Directive, a sperm bank in the EU must be licensed in order to operate. The Human Fertilization and Embryology Authority is responsible for overseeing sperm banks in the United Kingdom [5]. The sperm is cryogenically preserved in liquid nitrogen tanks after being treated for cryoprotection and placed in tiny vials or straws carrying between 0.4 and 1.0 mL of sperm.

Sperm may be preserved in two ways: traditional freezing or vitrification. The traditional method, employed mostly in assisted reproductive technologies (ARTs), entails a lengthy freezing procedure. However, vitrification is a more rapid technique of sperm cryopreservation since it does not require the removal of water. This method has the benefit of being faster for a "high cooling rate," but at the cost of increased contamination from liquid nitrogen and a lower sperm sample size [6]. Because it may be easily accessed with a common refrigerator, freeze-drying semen is yet another viable option for long-term storage. Animals have been used to test the effectiveness of this technique. However, DNA may be damaged in this process. Thus, further studies are necessary to understand the elements that might affect this method's success [7]. Artificial insemination and in vitro fertilization both involve the introduction of sperm into a female host. Standard artificial insemination is inserting sperm into the vagina via a catheter and depositing it at the cervix opening. For all intents and purposes, this is the identical biological mechanism that occurs in penile ejaculation where there is the release of sperm during sex. Due to its ease of use, this technique of insemination is often used for inseminations performed at home or by self, especially by women living alone or women in same-sex partnerships. Intrauterine insemination (IUI) and deep intrauterine artificial insemination are two forms of this technique and are two further applications that need "washed" sperm. When a woman has no underlying reproductive difficulties, these insemination procedures have been shown to have higher success rates than intracervical insemination (ICI), making them the gold standard in fertility clinics [8].

## Review

Freezing sperm: some technical considerations

Short-term preservation of semen in dry ice resulted in the first human live birth utilizing cryopreserved sperm in the year 1953 [9]. In 1963, long-term sperm cryopreservation using liquid nitrogen was demonstrated, and this development paved the way for sperm freezing to become known all over the world [10]. There was an assessment of a number of criteria thought to be important for the successful freezing of human spermatozoa [11]. Cryopreserving sperm for 40 years is safe and effective for use in fertility treatments [12]. There is a chance of viral contamination between sperm specimens kept frozen under liquid nitrogen [13]. As a result, nitrogen vapor is often used in contemporary sperm banks since it presumably poses a considerably reduced danger of viral cross-contamination [14]. Masturbation is the quickest and most convenient way to collect a semen sample for sperm banking, but there are other options as well. Vibratory penile stimulation devices that can be thrown away are easy to use and do not cause any harm to the user [15]. Because of the need for general anesthesia, electroejaculation is reserved for situations when the ejaculatory reflex arc has been disrupted, such as in spinal cord injuries [16]. When a poor sperm output is expected or when electroejaculation is unavailable, sperm retrieval surgeries, such as dermal sperm extraction and testicular sperm retrieval, might be useful in certain cases [17]. Studies have shown that the motility and viability of sperm may be accurately predicted by their properties before freezing [18]. However, hydrodynamic and oxidant pressure, poisonous effects from the cryoprotectant, and the development of ice crystals within the cell cause harm to sperm during the freezing and thawing process [19]. For instance, repeated cycles of freezing and thawing might decrease the amount of viable sperm [20]. So, the hazards of harm to sperm during cryopreservation must be managed. Most typically, glycerol is used with egg yolk to alleviate osmotic stress in spermatozoa [21]. Additional cryoprotective substances, such as zinc [22], resveratrol and ascorbic acid, and lactic acid, have all been studied to sustain the viability of frozen sperm. After being frozen, sperm might lose some of its functionality due to the formation of volatile oxygen species [23]. The effects of reactive oxygen species (ROS) may be mitigated with the use of antioxidants such as the TAT-peroxiredoxin-2 fusion protein [24]. This was found to reduce the harmful effects of ROS. The application of chemicals to cryopreserved sperm is an area that needs more exploration so that the chemicals can be used for common practices. So many research studies have looked at whether or not improved sperm selection methods may enhance post-thaw semen quality, with most of the attention being paid to selecting sperm by hyaluronic acid binding [25]. Nonetheless, a recent Cochrane review was not able to endorse their regular usage. Washing spermatozoa to remove seminal plasma before freezing has been shown to increase motility once sperm has been thawed [26]. When it comes to human immunodeficiency virus (HIV)-positive individuals, sperm washing is recommended as routine therapy by the American Society for Reproductive Medicine. Since 1999, one institution in the United Kingdom has been washing sperm samples from HIV-positive individuals. Yet, there have been no reports of seroconversions in births resulting from these samples [27]. To remove HIV from the seminal plasma and other non-sperm cells such as leukocytes, a number of sperm-washing methods are available. The more commonly used method among them is semen centrifugation in a 45-90% colloidal silica density gradient.

Danger factors of fertility loss in cancer patients

The most common reason to bank sperm is for the treatment of cancer. Higher rates of male infertility are associated with certain tumor types, including testicular cancer, Hodgkin's lymphoma, and leukemia. Recovery of spermatogenesis following gonadotoxic therapy for testicular cancer is possible in 50% of patients after two years [28]. However, rates of recovery vary widely depending on the cancer type, treatment strategy, and underlying testicular function. Infertility was reported by 46% of childhood cancer survivors in the Childhood Cancer Survivor study, compared to just 17.5% of their siblings who had also survived the disease. Significant risk factors for infertility were identified in the same research as bleomycin therapy [29], testicular radiation dosage 4 Gy, alkylating dose score 3, and surgical resection of any organ in the genital tract. Table 1 summarizes some variables that may affect a man's fertility following cancer treatment. As a result, more and more men are being treated for their cancer using biological treatments. There is some concern that mTOR inhibitors like everolimus may have an adverse effect on gonadal function. Imatinib, a tyrosine kinase inhibitor, has been linked to sperm defects in case reports and in vitro investigations [30]. Counseling young boys with chronic myeloid leukemia on the potential impact of tyrosine kinase inhibitor treatment on long-term fertility is thus recommended by the American Society of Clinical Oncology [31]. To know to what degree new biological agents provide an indication for sperm freezing in men with cancer, further investigation is needed.

**Table 1:** Sperm Collection Methods For Patients with Different Diseases

Patient Condition	Sperm Collection Method	What Makes Them Eligible for Sperm Banking?
Adult cancer	Frequent ejaculation	Cancer and related therapies damage the gonads and impair spermatogenesis
Adolescent cancer	Frequent ejaculation (when they are sexually mature), penile vibratory stimulation, and electroejaculation	Same as above
Cancer in prepubescent males	Testicular tissue freezing	Cancer treatment negatively affects spermatogenesis; testicular tissue extraction is a promising experimental procedure
Preoperative surgical procedure to treat or induce infertility	Frequent ejaculation	Bilateral varicocele ligation, prior to vasectomy
Non-malignant disease	Frequent ejaculation	Systemic stress may impair spermatogenesis; gonadotoxic therapies affect semen quality
Occupational risk	Frequent ejaculation	Exposure to harmful chemicals may decrease fertility or cause chromosomal damage in the germ cells; facing hazardous situations that may result in an accident that causes infertility
Posthumous sperm cryopreservation	Electroejaculation surgical removal of testes and epididymides	Performed at the time of brain death based upon the patient's will or family request
Sex change/ gender reassignment	Frequent ejaculation removal of testicular tissue	Hormone therapy damages spermatogenesis, and gender reassignment surgery sterilizes the male

Sperm banking, both autologous and donor, to treat infertility

By now, sperm freezing is well established as a possible course of action for infertile couples, and its therapeutic uses are fast expanding. Patients with severe oligospermia who are undergoing intracytoplasmic sperm injection (ICSI) often have their own sperm frozen as a backup sample. If sperm retrieval for ARTs like IUI, ICI, etc. cannot be performed at the same time as egg retrieval, the sperm may be frozen after epididymal sperm aspiration or testicular biopsy. If the patient is unable to produce healthy sperm, a sperm donor may be able to help. Other possible uses for donated sperm include treatment of infertility in unmarried women or women in same-sex partnerships and prevention of the vertical transmission of infectious or genetic diseases [32]. Before a man may donate sperm, he must first pass a comprehensive clinical evaluation. Serum blood tests are used to rule out infectious diseases like HIV, hepatitis B and C, syphilis, human T-lymphotropic virus, and cytomegalovirus. With the rise of new viruses like Ebola and Zika, infected individuals may spread the virus to their sexual partners even if they are showing no symptoms themselves [33]. Sperm banks must adapt their policies frequently to ensure that specific sperm donor populations are being screened adequately for viral pathogens [34]. Although the authors do not advocate for its frequent usage, pre-screening history and questionnaires may help determine whether or not genetic testing of sperm donors is warranted. An increasing frequency of abnormalities in sperm genomes and embryos generated by ARTs utilizing donor sperm have been connected to advancing age [35]. Therefore, the American Society for Reproductive Medicine (ASRM) and the Society for Assisted Reproductive Technology both state that sperm donors should ideally be under the age of 40. The official website of the Human Fertilization and Embryology Authority of the United Kingdom furthermore suggests a maximum age of 41 for sperm donors [36]. A recent meta-analysis, however, suggests that in an oocyte-donation model, there is no correlation between the paternal age and a lower chance of a successful pregnancy, a miscarriage, or a live delivery. In conclusion, heterosexual, gay, and transgender couples should preferably use sperm from donors less than the age of 40.

Potential for future fertility maintenance through conception by either a male partner or a secret donor

Once puberty hits, the ejaculate will include mature sperm. Cryopreservation is easiest in boys over the age of 13, according to a recent research study of 12-17-year-old males [37], although sperm may be obtained from any boy who has reached puberty. Since prepubescent males with cancer have a higher chance of survival than they had a decade ago, it is crucial to study whether or not fertility can be preserved in this population. Slow freezing with cryoprotectants such as 1.5 M dimethylsulfoxide and 0.15 M sucrose allows for the long-term storage of spermatogonial stem cells (SSCs) or testicular tissue. Ongoing research is looking at the feasibility of using frozen SSCs or testicular tissue samples to reestablish fertility. To maintain their stem cell capabilities, SSCs may be grown in vitro in a medium similar to that used for human embryonic stem cells. Male mice made infertile by busulfan treatment may conceive again after receiving an injection of an SSC suspension. One research study showed that 36.5% of infertile mice that were injected with an SSC solution into their testicles restored normal spermatogenesis. Additionally, spermatozoa may be adolescent, and prepubertal rhesus monkeys that have undergone sterilization by alkylating chemotherapy and subsequent injection of SSCs further into rete testes have sperm that can be detected in their ejaculate which fertilized oocytes when injected intracytoplasmically (ICSI). Together, these findings indicate that autotransplantation of SSCs may one day be used as a therapeutic option for treating cancer in boys. Nevertheless, there are still some mysteries to be solved: Will there be a chance that reintroduction of cancerous cells occurs if SSCs are transplanted back into the patient? How stable are SSCs epigenetically after cryopreservation [38]? It has been claimed that fluorescence-activated cell sorting may reduce the reintroduction of malignancies in SSC transplants. In the event that a patient is infertile due to cancer treatment before puberty, autologous testicular tissue transplantation might be a viable option. In prepubescent monkeys, transplanting testicular tissue led to complete spermatogenesis, although autograft survival was only 5%. Therefore, it seems that the success rate of grafts utilizing SSC injection is higher than that using testicular tissue transplantation. Autotransplantation of testicular tissue carries the potential danger of re-inoculating the patient with cancer cells; this risk must be reduced with stringent methods to assure safety. Thus, SSCs and testicular tissue are novel technologies with potential to expand the advantages of fertility preservation in prepubescent males in the future. Additional clinical trials are needed to verify their effectiveness and safety before they can be recommended for widespread usage [39].

Transgender individuals relying on sperm banks

A recent meta-analysis of data from various demographics found that the number of transgender individuals per 100,000 is 4.6. The number of transsexual individuals who undergo a transition from female to male is around three times higher than from male to female. Trans women are rendered temporarily or permanently sterile with the use of hormone treatment and/or bilateral orchiectomy. But with the right reproductive therapy, many transgender people can have children in the future. This is why the Endocrine Society says that individuals undergoing gender reassignment should be provided reproductive counseling [40]. In addition, the ASRM ethics committee suggests providing all persons undergoing gender reassignment with the option of gamete freezing. However, more studies are required to determine the efficacy of sperm banking as given by medical experts and employed by people for ARTs. To sum up, sperm banking is becoming an integral part of medical procedures for changing one's gender. Even though more and more physicians have insufficient awareness of this innovative therapeutic route, upcoming research is required to build clinical pathways to treat people who are interested in undergoing this procedure. A patient who is unable to ejaculate may need to undergo a sperm retrieval and freezing procedure. Reduction in sperm quality, inability to ejaculate, and impotence are all symptoms of a spinal cord injury. There is some evidence that abdominal and pelvic procedures, such as retroperitoneal lymph node dissection, aorto-iliac reconstruction, and colorectal excision, might lead to a loss of ejaculatory function. It has been estimated that as many as one-third of people with diabetes mellitus have retrograde ejaculation [41]. Multiple sclerosis is another medical condition associated with a high prevalence of ejaculatory dysfunction (50-75%). In one-third of instances, surgical sperm retrieval for ARTs is unnecessary if sympathomimetic drugs are used to treat retrograde ejaculation caused by surgery or diabetes. In addition to cancer, non-malignant illnesses such inflammatory bowel disease and glomerulonephritis may potentially benefit from sperm freezing before undergoing cytotoxic therapy. Though sperm freezing has been studied for various disorders, there is a lack of evidence assessing clinical effects.

## Conclusions

Sperm banking is a tried-and-true method for helping infertile couples to give birth and protecting the ability to reproduce when it is at risk. To guarantee that cancer patients get timely referrals to sperm banks, there has to be stronger communication between doctors and sperm bank providers. Another factor to think about is how expanding patient access to sperm banks can affect their treatment budgets. While cancer treatment is the most common reason to preserve fertility, there are many other conditions, risk factors, and treatments that could have an impact on sperm function as well. Therefore, it is crucial for all medical workers to ask their patients if they want to have children in the future.
